# 2-(Carboxy­methyl­sulfan­yl)pyridine-3-carboxylic acid monohydrate

**DOI:** 10.1107/S1600536810016120

**Published:** 2010-05-08

**Authors:** Xiao-Juan Wang, Yun-Long Feng

**Affiliations:** a Zhejiang Key Laboratory for Reactive Chemistry on Solid Surfaces, Institute of Physical Chemistry, Zhejiang Normal University, Jinhua, Zhejiang 321004, People’s Republic of China

## Abstract

The title compound, C_8_H_7_NO_4_S·H_2_O, was obtained by reaction of 2-mercaptopyridine-3-carboxylic acid with chloro­acetic acid. In the mol­ecular structure, the dihedral angle between the two least-squares planes defined by the pyridine ring and the carb­oxy group is 8.32 (9)°. The carboxy­methyl­sulfanyl group makes a torsion angle of 82.64 (12)° with the pyridine ring. An intra­molecular O—H⋯N hydrogen bond between the acidic function of the carboxy­methyl­sulfanyl group and the pyridine N atom stabilizes the conformation, whereas inter­molecular O—H⋯O hydrogen bonding with the uncoordinated water mol­ecules is responsible for packing of the structure, leading to chains propagating in [001].

## Related literature

For derivatives of 2-mercaptopyridine-3-carboxylic acid and compounds with 2-mercaptopyridine-3-carboxyl­ate ligands, see: Panagiotis *et al.* (2003 ▶); Smith & Sagatys (2003 ▶); Humphrey *et al.* (2006 ▶); Ma *et al.* (2004 ▶); Quintal *et al.* (2002 ▶).
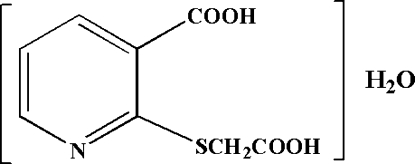


## Experimental

### 

#### Crystal data

C_8_H_7_NO_4_S·H_2_O
*M* *_r_* = 231.22
Triclinic, 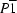

*a* = 7.2824 (2) Å
*b* = 7.3132 (2) Å
*c* = 10.9090 (4) Å
α = 77.901 (2)°
β = 71.787 (2)°
γ = 62.590 (2)°
*V* = 488.43 (3) Å^3^
*Z* = 2
Mo *K*α radiation
μ = 0.33 mm^−1^
*T* = 296 K
0.48 × 0.43 × 0.04 mm

#### Data collection

Bruker APEXII CCD diffractometer
Absorption correction: multi-scan (*SADABS*; Sheldrick, 1996 ▶) *T*_min_ = 0.853, *T*_max_ = 0.987
7375 measured reflections
2217 independent reflections
1910 reflections with *I* > 2σ(*I*)
*R*_int_ = 0.024

#### Refinement

*R*[*F* ^2^ > 2σ(*F* ^2^)] = 0.032
*wR*(*F* ^2^) = 0.090
*S* = 1.02
2217 reflections
148 parameters
5 restraints
H atoms treated by a mixture of independent and constrained refinement
Δρ_max_ = 0.24 e Å^−3^
Δρ_min_ = −0.24 e Å^−3^

### 

Data collection: *APEX2* (Bruker, 2006 ▶); cell refinement: *SAINT* (Bruker, 2006 ▶); data reduction: *SAINT*; program(s) used to solve structure: *SHELXS97* (Sheldrick, 2008 ▶); program(s) used to refine structure: *SHELXL97* (Sheldrick, 2008 ▶); molecular graphics: *DIAMOND* (Crystal Impact, 2008 ▶); software used to prepare mat­erial for publication: *SHELXTL* (Sheldrick, 2008 ▶).

## Supplementary Material

Crystal structure: contains datablocks I, global. DOI: 10.1107/S1600536810016120/wm2333sup1.cif

Structure factors: contains datablocks I. DOI: 10.1107/S1600536810016120/wm2333Isup2.hkl

Additional supplementary materials:  crystallographic information; 3D view; checkCIF report

## Figures and Tables

**Table 1:** Hydrogen-bond geometry (Å, °)

*D*—H⋯*A*	*D*—H	H⋯*A*	*D*⋯*A*	*D*—H⋯*A*
O1*W*—H1*WA*⋯O3^i^	0.83 (2)	2.02 (2)	2.8319 (18)	166 (2)
O1*W*—H1*WB*⋯O3^ii^	0.83 (2)	1.98 (2)	2.7784 (18)	162 (2)
O1—H1⋯O1*W*^iii^	0.83 (2)	1.76 (2)	2.5917 (17)	171 (2)
O4—H4⋯N1	0.86 (2)	1.72 (2)	2.5778 (17)	172 (2)

Symmetry codes: (i) 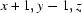; (ii) 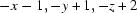; (iii) 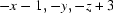.

**Fig. 1.**
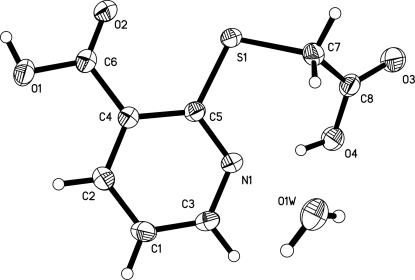
View of the molecular structure of (I), showing the atom-numbering scheme. Displacement ellipsoids are drawn at the 30% probability level.

**Fig. 2.**
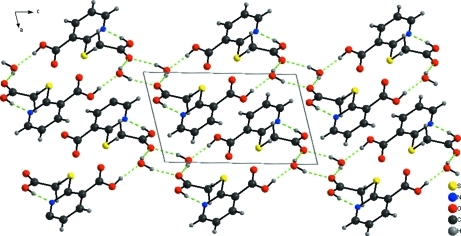
A view of the one-dimensional chain structure of (I). Intermolecular hydrogen bonds interactions are depicted by dashed lines.

## supplementary crystallographic information

### Comment

2-Mercaptopyridine-3-carboxylic acid is an interesting ligand because of its potential versatile coordinate behavior. It may act as a deprotonated ligand through either the carboxylate or the thiolate group, such as 2-mercaptopyridine-3-carboxylate hydrate (Smith *et al.*, 2003) or 2-mercapto-nicotinic acid (Panagiotis *et al.*, 2003). Thus it can act as a monodentate (O or N), bidentate (O, O or O, N) or chelating (O, O or O, N) ligand interacting with metal ions, and a variety of coordination polymers have been characterised (Humphrey *et al.*, 2006; Ma *et al.*, 2004; Quintal *et al.*, 2002). In this work, we report a new derivative, 2-(carboxymethylsulfanyl)pyridine-3-carboxylic acid, (I), which was obtained by reaction of 2-mercaptopyridine-3-carboxylic acid with chloroacetic acid.

The molecular structure of (I) is presented in Fig. 1. The carboxylate group is almost parallel to the pyridine group with a dihedral angle of 8.32 (9)°, while the carboxymethylsulfanyl group makes a torsion angle of 82.64 (12)° with the pyridine ring. The carboxylic O atoms, pyridine N atom together with lattice water molecules are involved in hydrogen-bonding interactions (Fig. 2). In detail, the structure is stabilized by an intramolecular O—H···N hydrogen bond between the carboxy function of the carboxymethylsulfanyl group and the pyridine N atom. The other carboxy function acts as a donor and acceptor group for intermolecular O—H···O hydrogen bonds with the adjacent lattice water molecules which results in the formation of a chain structure running along the *c* direction.

### Experimental

The mixture of 2-mercaptopyridine-3-carboxylic acid (1.552 g, 10.0 mmol) and chloroacetic acid (2.835 g, 30.0 mmol) was stirred and refluxed under basic condition in which sodium hydroxide solution was needed to keep the pH around 11. After reaction for 4 h at 328 K, the mixture was cooled to room temperature. By adjusting the pH around 3 with concentrated hydrochloric acid, a white precipitate appeared rapidly. The solid was filtered off and washed with water. Single crystals suitable for X-ray diffraction were obtained in the mother liquid after evaporation within a few days.

### Refinement

The carbon-bound H-atoms were positioned geometrically and included in the refinement using a riding model [C—H 0.93, 0.97 Å U_iso_(H) = 1.2U_eq_(C)]. The oxygen-bound H-atoms were located in a difference Fourier maps and refined with an O—H distance restraint of 0.83 Å [U_iso_(H) = 1.2U_eq_(O)].

### Crystal data

**Table:**

C_8_H_7_NO_4_S·H_2_O	*Z* = 2
*M**_r_* = 231.22	*F*(000) = 240
Triclinic, *P*1	*D*_x_ = 1.572 Mg m^−^^3^
Hall symbol: -P 1	Mo *K*α radiation, λ = 0.71073 Å
*a* = 7.2824 (2) Å	Cell parameters from 3676 reflections
*b* = 7.3132 (2) Å	θ = 2.0–27.6°
*c* = 10.9090 (4) Å	µ = 0.33 mm^−^^1^
α = 77.901 (2)°	*T* = 296 K
β = 71.787 (2)°	Sheet, colourless
γ = 62.590 (2)°	0.48 × 0.43 × 0.04 mm
*V* = 488.43 (3) Å^3^	

### Data collection

**Table:**

Bruker APEXII CCD diffractometer	2217 independent reflections
Radiation source: fine-focus sealed tube	1910 reflections with *I* > 2σ(*I*)
graphite	*R*_int_ = 0.024
ω scans	θ_max_ = 27.6°, θ_min_ = 2.0°
Absorption correction: multi-scan (*SADABS*; Sheldrick, 1996)	*h* = −9→9
*T*_min_ = 0.853, *T*_max_ = 0.987	*k* = −9→9
7375 measured reflections	*l* = −14→14

### Refinement

**Table:**

Refinement on *F*^2^	Primary atom site location: structure-invariant direct methods
Least-squares matrix: full	Secondary atom site location: difference Fourier map
*R*[*F*^2^ > 2σ(*F*^2^)] = 0.032	Hydrogen site location: inferred from neighbouring sites
*wR*(*F*^2^) = 0.090	H atoms treated by a mixture of independent and constrained refinement
*S* = 1.02	*w* = 1/[σ^2^(*F*_o_^2^) + (0.0509*P*)^2^ + 0.088*P*] where *P* = (*F*_o_^2^ + 2*F*_c_^2^)/3
2217 reflections	(Δ/σ)_max_ = 0.001
148 parameters	Δρ_max_ = 0.24 e Å^−^^3^
5 restraints	Δρ_min_ = −0.24 e Å^−^^3^

### Special details

**Table:**

Geometry. All esds (except the esd in the dihedral angle between two l.s. planes) are estimated using the full covariance matrix. The cell esds are taken into account individually in the estimation of esds in distances, angles and torsion angles; correlations between esds in cell parameters are only used when they are defined by crystal symmetry. An approximate (isotropic) treatment of cell esds is used for estimating esds involving l.s. planes.
Refinement. Refinement of *F*^2^ against ALL reflections. The weighted *R*-factor wR and goodness of fit *S* are based on *F*^2^, conventional *R*-factors *R* are based on *F*, with *F* set to zero for negative *F*^2^. The threshold expression of *F*^2^ > σ(*F*^2^) is used only for calculating *R*-factors(gt) etc. and is not relevant to the choice of reflections for refinement. *R*-factors based on *F*^2^ are statistically about twice as large as those based on *F*, and *R*- factors based on ALL data will be even larger.

### Fractional atomic coordinates and isotropic or equivalent isotropic displacement parameters (Å^2^)

**Table:**

	*x*	*y*	*z*	*U*_iso_*/*U*_eq_	
S1	−0.82738 (6)	0.36814 (5)	1.36087 (3)	0.04015 (13)	
N1	−0.5286 (2)	0.09443 (18)	1.19465 (11)	0.0391 (3)	
O2	−0.92675 (19)	0.12020 (17)	1.57117 (11)	0.0515 (3)	
O4	−0.6871 (2)	0.41454 (18)	1.04260 (11)	0.0545 (3)	
C5	−0.6485 (2)	0.1157 (2)	1.31717 (13)	0.0336 (3)	
O1	−0.7165 (2)	−0.20956 (19)	1.61482 (11)	0.0523 (3)	
C4	−0.6261 (2)	−0.0580 (2)	1.40665 (13)	0.0344 (3)	
O3	−0.8462 (2)	0.74565 (18)	1.07003 (11)	0.0612 (4)	
C6	−0.7717 (2)	−0.0376 (2)	1.53873 (14)	0.0381 (3)	
C3	−0.3782 (3)	−0.0926 (2)	1.15876 (15)	0.0446 (4)	
H3A	−0.2974	−0.1047	1.0734	0.054*	
C8	−0.7529 (2)	0.5679 (2)	1.11102 (15)	0.0431 (3)	
C7	−0.7046 (3)	0.5195 (2)	1.24178 (14)	0.0408 (3)	
H7A	−0.7496	0.6490	1.2771	0.049*	
H7B	−0.5511	0.4459	1.2295	0.049*	
C2	−0.4654 (2)	−0.2483 (2)	1.36726 (15)	0.0412 (3)	
H2A	−0.4430	−0.3646	1.4256	0.049*	
C1	−0.3389 (3)	−0.2664 (2)	1.24242 (16)	0.0461 (4)	
H1A	−0.2298	−0.3931	1.2158	0.055*	
O1W	−0.0210 (2)	0.1439 (2)	1.15804 (12)	0.0589 (3)	
H1WA	0.034 (3)	0.020 (2)	1.145 (2)	0.071*	
H1WB	−0.081 (3)	0.200 (3)	1.0980 (19)	0.071*	
H4	−0.626 (3)	0.302 (3)	1.087 (2)	0.071*	
H1	−0.809 (3)	−0.177 (3)	1.6845 (18)	0.071*	

### Atomic displacement parameters (Å^2^)

**Table:**

	*U*^11^	*U*^22^	*U*^33^	*U*^12^	*U*^13^	*U*^23^
S1	0.0438 (2)	0.0341 (2)	0.0296 (2)	−0.00903 (16)	−0.00324 (15)	−0.00379 (14)
N1	0.0419 (7)	0.0382 (6)	0.0309 (6)	−0.0151 (5)	−0.0021 (5)	−0.0057 (5)
O2	0.0511 (7)	0.0457 (6)	0.0380 (6)	−0.0125 (5)	0.0027 (5)	−0.0045 (5)
O4	0.0775 (9)	0.0477 (7)	0.0319 (6)	−0.0222 (6)	−0.0154 (6)	0.0017 (5)
C5	0.0334 (7)	0.0354 (7)	0.0301 (7)	−0.0134 (6)	−0.0065 (5)	−0.0043 (5)
O1	0.0528 (7)	0.0501 (6)	0.0378 (6)	−0.0165 (6)	−0.0070 (5)	0.0093 (5)
C4	0.0351 (7)	0.0357 (7)	0.0326 (7)	−0.0158 (6)	−0.0080 (6)	−0.0020 (5)
O3	0.0693 (8)	0.0445 (7)	0.0426 (6)	−0.0061 (6)	−0.0137 (6)	0.0073 (5)
C6	0.0417 (8)	0.0420 (8)	0.0328 (7)	−0.0206 (7)	−0.0102 (6)	0.0008 (6)
C3	0.0426 (8)	0.0450 (8)	0.0376 (8)	−0.0160 (7)	0.0029 (6)	−0.0121 (6)
C8	0.0408 (8)	0.0432 (8)	0.0352 (8)	−0.0148 (7)	−0.0052 (6)	0.0031 (6)
C7	0.0482 (9)	0.0333 (7)	0.0376 (7)	−0.0147 (6)	−0.0123 (7)	−0.0001 (6)
C2	0.0428 (8)	0.0345 (7)	0.0437 (8)	−0.0154 (6)	−0.0103 (7)	−0.0011 (6)
C1	0.0414 (8)	0.0369 (8)	0.0504 (9)	−0.0107 (6)	−0.0025 (7)	−0.0124 (7)
O1W	0.0736 (9)	0.0588 (8)	0.0365 (6)	−0.0272 (7)	−0.0108 (6)	0.0050 (6)

### Geometric parameters (Å, °)

**Table:**

S1—C5	1.7606 (14)	O3—C8	1.2184 (18)
S1—C7	1.8151 (15)	C3—C1	1.368 (2)
N1—C3	1.3421 (19)	C3—H3A	0.9300
N1—C5	1.3438 (18)	C8—C7	1.507 (2)
O2—C6	1.2057 (18)	C7—H7A	0.9700
O4—C8	1.2949 (19)	C7—H7B	0.9700
O4—H4	0.861 (15)	C2—C1	1.379 (2)
C5—C4	1.4078 (19)	C2—H2A	0.9300
O1—C6	1.3180 (18)	C1—H1A	0.9300
O1—H1	0.834 (16)	O1W—H1WA	0.831 (15)
C4—C2	1.388 (2)	O1W—H1WB	0.830 (15)
C4—C6	1.487 (2)		
			
C5—S1—C7	101.30 (7)	O3—C8—O4	121.27 (15)
C3—N1—C5	119.77 (13)	O3—C8—C7	121.00 (15)
C8—O4—H4	108.4 (15)	O4—C8—C7	117.70 (13)
N1—C5—C4	120.67 (13)	C8—C7—S1	116.34 (11)
N1—C5—S1	117.29 (10)	C8—C7—H7A	108.2
C4—C5—S1	122.02 (11)	S1—C7—H7A	108.2
C6—O1—H1	103.5 (16)	C8—C7—H7B	108.2
C2—C4—C5	117.92 (13)	S1—C7—H7B	108.2
C2—C4—C6	121.27 (13)	H7A—C7—H7B	107.4
C5—C4—C6	120.81 (13)	C1—C2—C4	120.56 (14)
O2—C6—O1	123.86 (14)	C1—C2—H2A	119.7
O2—C6—C4	122.82 (13)	C4—C2—H2A	119.7
O1—C6—C4	113.31 (13)	C3—C1—C2	118.13 (14)
N1—C3—C1	122.77 (14)	C3—C1—H1A	120.9
N1—C3—H3A	118.6	C2—C1—H1A	120.9
C1—C3—H3A	118.6	H1WA—O1W—H1WB	101.9 (18)
			
C3—N1—C5—C4	−3.5 (2)	C2—C4—C6—O1	7.13 (19)
C3—N1—C5—S1	174.95 (11)	C5—C4—C6—O1	−173.44 (13)
C7—S1—C5—N1	−23.50 (12)	C5—N1—C3—C1	−0.4 (2)
C7—S1—C5—C4	154.90 (12)	O3—C8—C7—S1	117.68 (15)
N1—C5—C4—C2	5.0 (2)	O4—C8—C7—S1	−64.05 (18)
S1—C5—C4—C2	−173.31 (11)	C5—S1—C7—C8	82.64 (12)
N1—C5—C4—C6	−174.42 (12)	C5—C4—C2—C1	−2.8 (2)
S1—C5—C4—C6	7.24 (18)	C6—C4—C2—C1	176.64 (13)
C2—C4—C6—O2	−171.59 (14)	N1—C3—C1—C2	2.6 (2)
C5—C4—C6—O2	7.8 (2)	C4—C2—C1—C3	−0.9 (2)

### Hydrogen-bond geometry (Å, °)

**Table:**

*D*—H···*A*	*D*—H	H···*A*	*D*···*A*	*D*—H···*A*
O1W—H1WA···O3^i^	0.83 (2)	2.02 (2)	2.8319 (18)	166 (2)
O1W—H1WB···O3^ii^	0.83 (2)	1.98 (2)	2.7784 (18)	162 (2)
O1—H1···O1W^iii^	0.83 (2)	1.76 (2)	2.5917 (17)	171 (2)
O4—H4···N1	0.86 (2)	1.72 (2)	2.5778 (17)	172 (2)

Symmetry codes: (i) *x*+1, *y*−1, *z*; (ii) −*x*−1, −*y*+1, −*z*+2; (iii) −*x*−1, −*y*, −*z*+3.
